# Evaluation of Mosquito Magnet and other collection tools for *Anopheles* mosquito vectors of simian malaria

**DOI:** 10.1186/s13071-021-04689-3

**Published:** 2021-04-01

**Authors:** Nantha Kumar Jeyaprakasam, Sandthya Pramasivan, Jonathan Wee Kent Liew, Lun Van Low, Wan-Yusoff Wan-Sulaiman, Romano Ngui, Jenarun Jelip, Indra Vythilingam

**Affiliations:** 1 Department of Parasitology, Faculty of Medicine, University of Malaya, Kuala Lumpur, Malaysia; 2 Tropical Infectious Diseases Research and Education Centre (TIDREC), University of Malaya, Kuala Lumpur, Malaysia; 3 Division of Disease Control, Ministry of Health Malaysia, Putrajaya, Malaysia

**Keywords:** *Anopheles*, Mosquito Magnet, *Plasmodium knowlesi*, Simian malaria, Trapping methods, Vector surveillance, Zoonosis

## Abstract

**Background:**

Vector surveillance is essential in determining the geographical distribution of mosquito vectors and understanding the dynamics of malaria transmission. With the elimination of human malaria cases, knowlesi malaria cases in humans are increasing in Malaysia. This necessitates intensive vector studies using safer trapping methods which are both field efficient and able to attract the local vector populations. Thus, this study evaluated the potential of Mosquito Magnet as a collection tool for *Anopheles* mosquito vectors of simian malaria along with other known collection methods.

**Methods:**

A randomized 4 × 4 Latin square designed experiment was conducted to compare the efficiency of the Mosquito Magnet against three other common trapping methods: human landing catch (HLC), CDC light trap and human baited trap (HBT). The experiment was conducted over six replicates where sampling within each replicate was carried out for 4 consecutive nights. An additional 4 nights of sampling was used to further evaluate the Mosquito Magnet against the “gold standard” HLC. The abundance of *Anopheles* sampled by different methods was compared and evaluated with focus on the *Anopheles* from the Leucosphyrus group, the vectors of knowlesi malaria.

**Results:**

The Latin square designed experiment showed HLC caught the greatest number of *Anopheles* mosquitoes (*n* = 321) compared to the HBT (*n* = 87), Mosquito Magnet (*n* = 58) and CDC light trap (*n* = 13). The GLMM analysis showed that the HLC method caught significantly more *Anopheles* mosquitoes compared to Mosquito Magnet (*P* = 0.049). However, there was no significant difference in mean nightly catch of *Anopheles* mosquitoes between Mosquito Magnet and the other two trapping methods, HBT (*P* = 0.646) and CDC light traps (*P* = 0.197). The mean nightly catch for both *An. introlatus* (9.33 ± 4.341) and *An. cracens* (4.00 ± 2.273) caught using HLC was higher than that of Mosquito Magnet, though the differences were not statistically significant (*P* > 0.05). This is in contrast to the mean nightly catch of *An. sinensis* (15.75 ± 5.640) and *An. maculatus* (15.78 ± 3.479) where HLC showed significantly more mosquito catches compared to Mosquito Magnet (*P* < 0.05).

**Conclusions:**

Mosquito Magnet has a promising ability to catch *An. introlatus* and *An. cracens*, the important vectors of knowlesi and other simian malarias in Peninsular Malaysia. The ability of Mosquito Magnet to catch some of the *Anopheles* mosquito species is comparable to HLC and makes it an ethical and safer alternative.

**Graphic Abstract:**

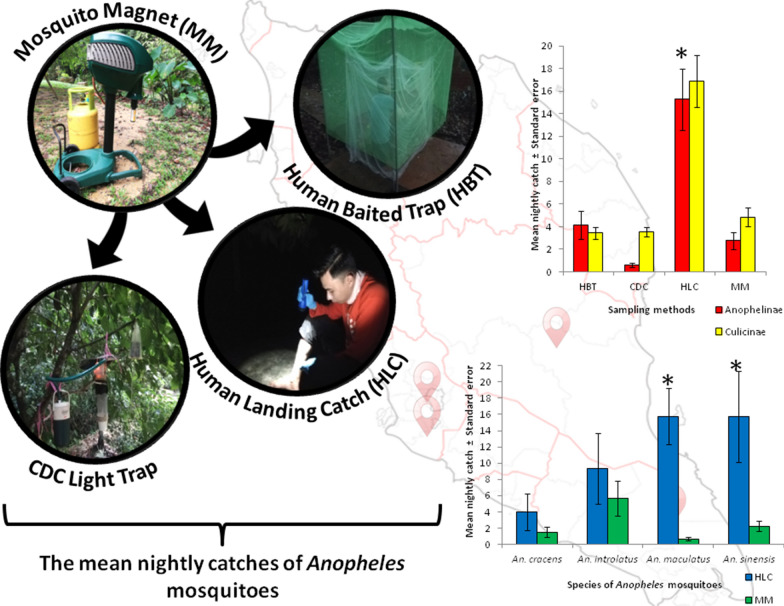

**Supplementary Information:**

The online version contains supplementary material available at 10.1186/s13071-021-04689-3.

## Background

Malaria continues to be a global public health problem especially in Africa [1]. In the Asia-Pacific region, about 2.2 billion people are at risk of malaria [2]. However, many countries have made progress toward malaria elimination, including Malaysia. Malaysia has shown great success in moving closer to its goal of eliminating indigenous human malaria transmission, evidenced by the number of locally acquired human malaria cases plummeting to zero in 2018 [3]. However, the ongoing increase in zoonotic *Plasmodium knowlesi* cases poses a major challenge to malaria control and might cause Malaysia to miss its goal to be a malaria-free country. Currently, knowlesi malaria is the predominant malaria infection in Malaysia [3, 4]. Although a significant proportion of the knowlesi malaria cases in Malaysia were previously confined to Malaysian Borneo, the increase in the number of cases in Peninsular Malaysia for the past few years from 113 cases in 2015 to 598 cases in 2018 is alarming [5].

Besides, more information on knowlesi malaria vectors is crucial to better understand the bionomics of the vectors and its role in malaria transmissions. The increased cases of knowlesi malaria in Malaysia [4] certainly demand proper vector surveillance to understand the transmission dynamics of simian *Plasmodium* to humans from its natural macaque hosts. In addition, reports on natural human infection with *Plasmodium cynomolgi* [6–9] and possibly other simian *Plasmodium* necessitate more intensive vector surveillance in Malaysia and the Southeast Asian region. Vector incrimination by gathering site-specific information on the vectors is an essential step in planning effective control measures [10].

Unfortunately, information on the distribution of the vectors of zoonotic simian malaria is still sparse in Malaysia [11]. Thus, it is essential to identify reliable adult mosquito sampling techniques which can characterize mosquito biting density on humans. Even though there are various methods for adult mosquito sampling, human landing catch (HLC) remains the “gold standard” [12]. HLC is the most reliable method to represent the human biting rate, but it is labor intensive and very risky especially when the collectors were exposed to infective mosquito bites while performing the catches. HLC exposes the participants to an array of vector-borne diseases such as chikungunya, dengue, malaria, filariasis and viral encephalitis, for many of which there is no prophylaxis or only limited treatment options [13]. The emergence of drug-resistant *Plasmodium falciparum* further exacerbates the health and ethical issues related to HLC in malaria endemic countries like in Southeast Asia. Besides mosquitoes, the participants in HLC are also exposed to other blood-feeding arthropods such as ticks which could cause Lyme disease [14]. Besides being very laborious, the mosquitoes collected through HLC are also influenced by the skills of the collectors and body odors which might affect the type and quantity of mosquitoes caught [15].

Since the *Anopheles* vectors of knowlesi malaria are forest-dwelling mosquitoes that belong to the Leucosphyrus group [16], the conventional trapping techniques such as HLC can be challenging. This includes the possibilities of encountering dangerous wild animals when sampling in deep forested area for long hours. Thus, lack of data on the spatiotemporal distribution of mosquito species in certain areas can impede the process of understanding the zoonotic transmission dynamics of simian malaria. Although there are other trapping methods that use physical and chemical attractants for vector surveillance (without using humans as bait), each method has its own limitations. In general, all trapping methods and attractants have variable performances compared to HLC for *Anopheles* mosquitoes sampling [17]. Considering that the current conventional approaches are challenged by many factors, an alternative trapping method is urgently needed for the surveillance of simian malaria vectors.

Mosquito Magnet may offer a novel solution to some of the issues associated with the conventional trapping methods. Mosquito Magnet was originally designed to catch and kill mosquitoes by dehydrating them in the net by trapping them for many days. However, when the mosquitoes are collected within the same day, live mosquitoes can be obtained, which can be dissected for further entomological investigation. Several studies have evaluated the efficiency of Mosquito Magnet in trapping mosquitoes [18–22]. However, all those studies were conducted in countries outside Southeast Asia where different species of *Anopheles* mosquitoes were caught. Mosquito Magnet was found to be effective in catching Neo-tropical *Anopheles* species, i.e. *An. nuneztovari* and *An. darlingi*, which significantly correlates with the results of HLC, but is not efficient in collecting *An. marajoara* [23]. These findings call for further investigations to assess the effectiveness of Mosquito Magnet for surveillance of *Anopheles* populations in Malaysia. Furthermore, the efficacy of the Mosquito Magnet in trapping *Anopheles* mosquitoes from the Leucosphyrus group, which are the known vectors for knowlesi and simian malaria, has not been determined while other methods such as CDC light traps [24] and human baited traps [25] have been previously evaluated.

Thus, this study aimed to assess the effectiveness of Mosquito Magnet in catching *Anopheles* mosquitoes along with other tools for entomological surveillance in Peninsular Malaysia. Since most of the knowlesi malaria cases occur in forested areas and forest fringes, it is pertinent to have alternative tools in view of the challenges posed by the conventional HLC sampling technique.

## Methods

### Study site

**Fig. 1:**
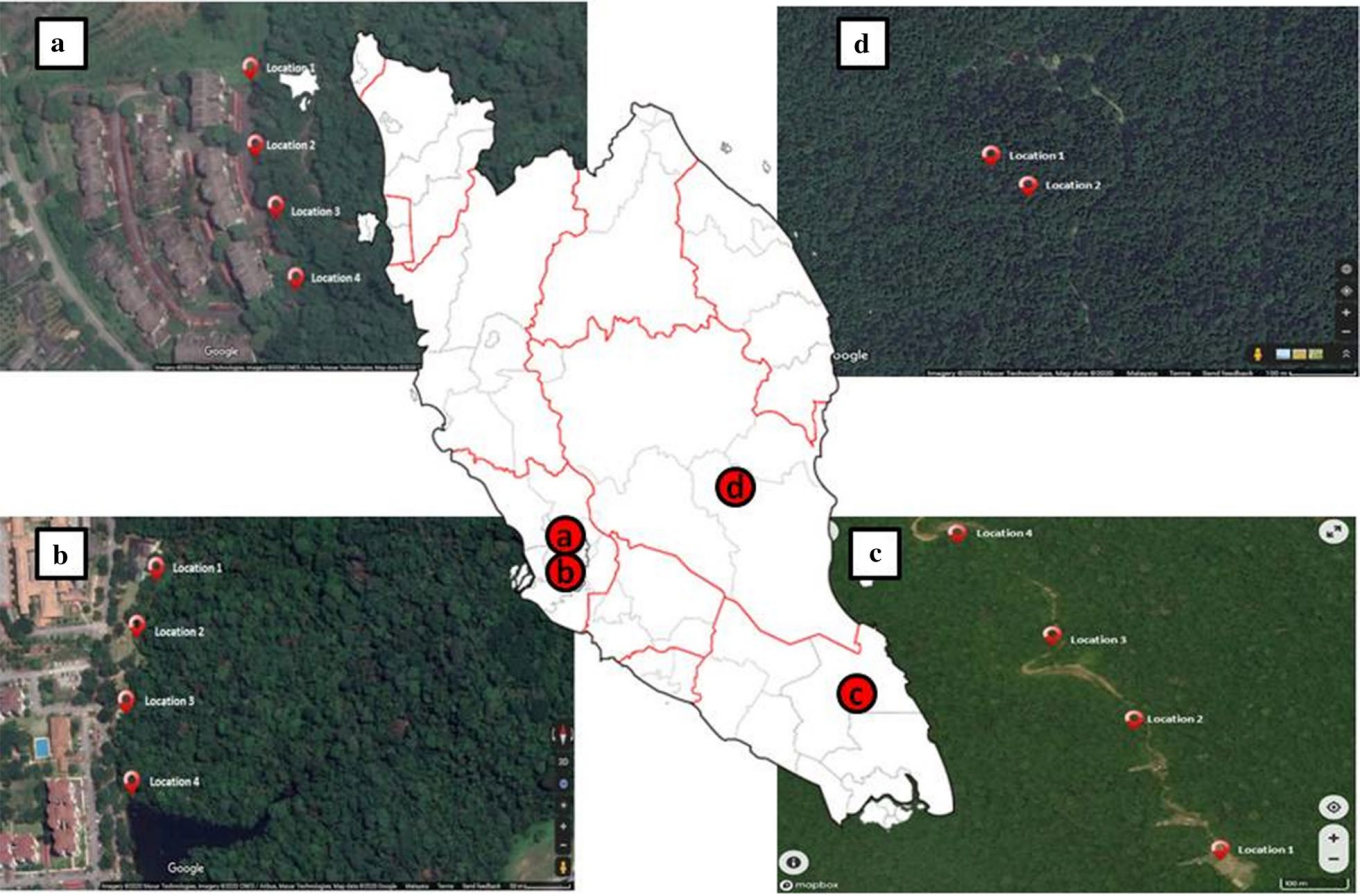
Map of Peninsular Malaysia showing the sampling location with respective sampling sites for the Latin square designed experiment: **a** Forest patch in Serendah, Selangor. **b** Community forest reserve in Kota Damansara, Selangor, and **c** dense forested area in Bukit Tinggi, Johor. Sampling location **d** A forest in Kem Sri Gading, Pahang, was also included to further compare the effectiveness of HLC and Mosquito Magnet trapping methods

The study was conducted in three different states in Peninsular Malaysia: Selangor, Johor and Pahang (Fig. 1). All locations were selected based on preliminary findings on the presence and density of *Anopheles* mosquitoes using HLC. Two different locations were selected in Selangor: a community forest reserve in Kota Damansara (3° 10′ 06.0″ N, 101° 34′ 50.7″ E) and a small forest patch in Serendah (3° 23′ 20.5″ N, 101°37′55.5″ E). Sampling was conducted at the forest fringes. Meanwhile, in Johor, a forested area in Bukit Tinggi (2° 17′ 14.1″ N, 103° 40′ 27.8″ E) was selected as the study location. It is a virgin forest situated in a hilly terrain where the lowest sampling site has an elevation of 80 m while the highest elevation is 325 m above sea level. In Pahang, the camping site Kem Sri Gading (3° 45′ 37.9″ N 102° 34′ 20.2″ E) at Jengka was chosen. In all four study locations, long-tailed macaques (*Macaca fascicularis*) were sighted. In addition, preliminary studies also indicated the presence of *Anopheles* from the Leucosphyrus group in both locations selected in Johor and Pahang. In addition, there were also human knowlesi malaria cases reported from both areas: at least three cases in Kem Microwave Bukit Tinggi, Johor, and one case in Kem Sri Gading Jengka, Pahang, from 2011 until 2019 (unpublished data from the Ministry of Health Malaysia).

### Study design

Mosquito collections were conducted using four methods, i.e. human landing catch (HLC), CDC light trap, human baited trap (HBT) and Mosquito Magnet, performed between November 2019 and July 2020 based on the randomized 4 × 4 Latin square design. The experiment was conducted with six replicates where sampling within each replicate occurred on 4 consecutive nights. Unfortunately, the sampling duration was reduced to five replicates because of travel restrictions due to the unprecedented COVID-19 pandemic. Sampling sites for the Latin square design were between 80 and 200 m apart (Fig. 1), and the methods were rotated among the four sites each night to minimize the effect of site variation. The collections were conducted between 1800 and 0000 h. Further comparative evaluation between the Mosquito Magnet and the “gold standard” HLC was performed between August 2020 and October 2020 over 4 nights in Kem Sri Gading.

### Trapping methods

#### CDC light trap

**Fig. 2:**
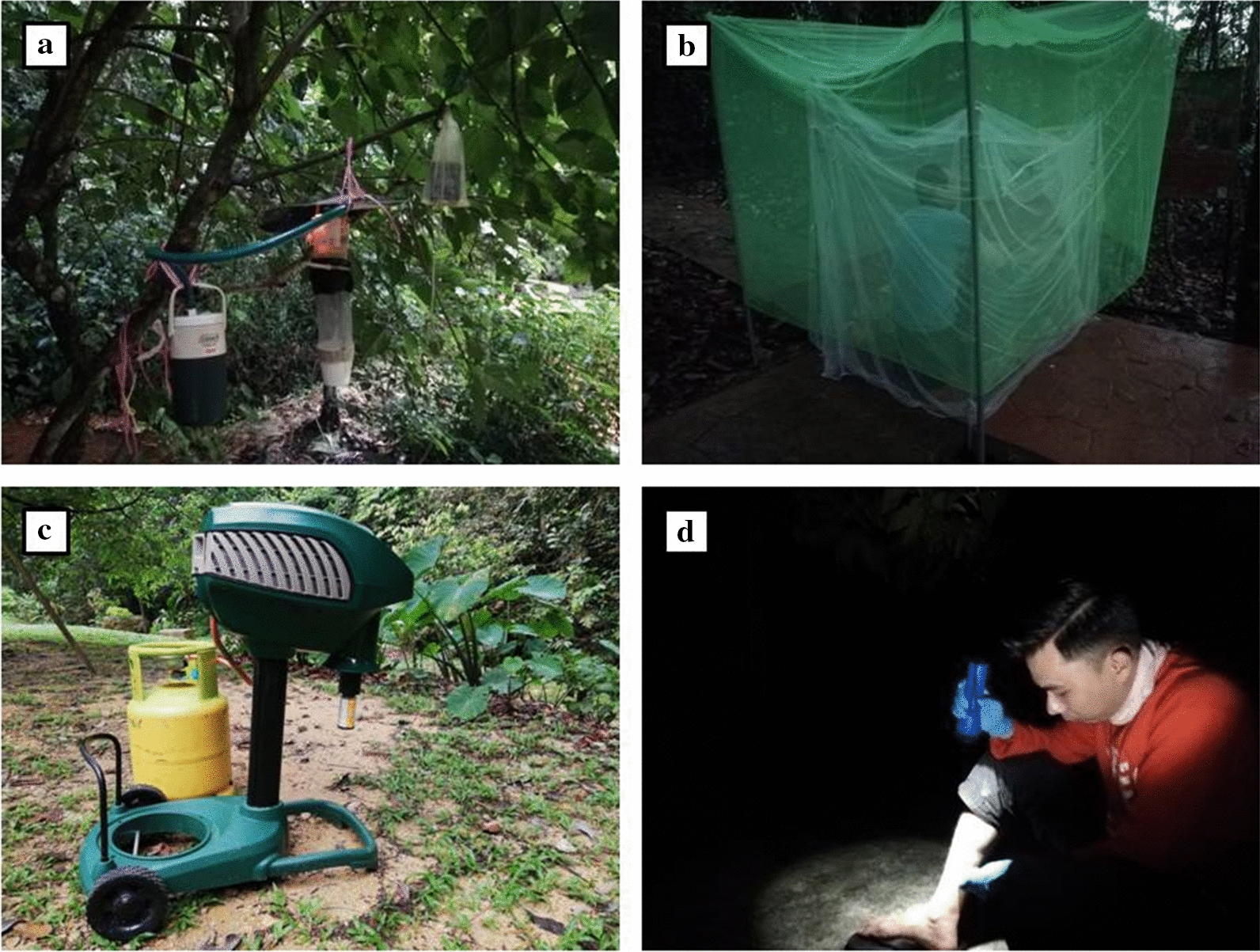
Trapping methods. **a** CDC light trap. **b** Human baited trap (HBT). **c** Mosquito Magnet (MM). **d** Human landing catch (HLC)

A CDC light trap fitted with an incandescent bulb was suspended from trees around 1.5 m above ground. Mosquitoes attracted to the trap were drawn by a 6-V (6Ah) battery-powered fan in the collection container. Carbon dioxide (CO_2_) was produced by the sublimation of dry ice in a clean thermo-flask, which was hung adjacent to the light trap. The CO_2_ produced from 1 kg dry ice was passed through tubing (2 cm diameter), and the point of emission was placed above the suction fan and below the metal cover (Fig. 2a).

#### Human-baited trap (HBT)

Two adults were seated on small stools inside a small, fully protected, untreated white polyester bed net (150 cm high × 165 cm long × 100 cm wide, mesh size 1.5 mm), which was hung until it touched the ground. A larger green untreated polyester bed net (175 cm high × 230 cm long × 175 cm wide, mesh size 1.5 mm) was hung over the smaller net, leaving 15–20 cm between the ground and the lower edge of the net (Fig. 2b). Mosquitoes were caught between the two nets. The mosquitoes were collected for 10 min at the end of each hour from 1800 until 0000 h. The participants pulled the outer net to the ground, came out from the inner net and aspirated the mosquitoes, which were trapped between the two nets. The aspirated mosquitoes were transferred into different cups each hour.

#### Mosquito Magnet (MM)

The Mosquito Magnet (Model: Independence; Manufacturer: Woodstream Corp., USA) uses Lurex^3^ attractant, carbon dioxide and counterflow technology to capture the mosquitoes. It was assembled and operated as per the manufacturer’s instructions (Fig. 2c). Proper placement of the Mosquito Magnet is crucial for its functionality. The Mosquito Magnet was placed facing the potential mosquito breeding site. Through a catalytic conversion process of the propane gas, the mosquito magnet emits a plume of carbon dioxide (CO_2_), heat and moisture from the inner attractant tube, while the flared outer tube vacuums the mosquitoes along the top of the CO_2_ plume without vacuuming up any of the CO_2_ [22, 26, 27]. The propane gas required by the Mosquito Magnet was supplied by a gas cylinder. The Lurex^3^ attractant cartridge used in the Mosquito Magnet contains a Lurex component (lactic acid 35.4%) and ammonium bicarbonate component (ammonium bicarbonate 74.63%). The Mosquito Magnet is powered by four 1.5-V batteries. As with the other traps, the Mosquito Magnet was operated between 1800 and 0000 h. The collection net from the Mosquito Magnet was emptied at the end of the night, and all the mosquitoes were sorted and identified.

#### Human landing catch (HLC)

HLC was performed by a pair of trained collectors from 1800 until 0000 h each night. The same pair of collectors performed the HLC each night to prevent collection bias. All mosquitoes that landed on the bare legs were caught using 50 × 19 mm glass vials (Fig. 2d). The glass vials contained a small damp tissue at the base to keep the environment inside the vial humid. Once the mosquito was trapped, the vial was plugged with cotton wool. The mosquitoes were separated based on the hours of collection.

### Mosquito identification

After collection, the mosquitoes were carefully sorted according to the genus on site. *Anopheles* mosquitoes were identified to species level the next day. The *Anopheles* mosquitoes were morphologically identified using the keys of Reid (1968) [28] while the keys of Sallum (2005) [29] were used for the identification of the Leucosphyrus group. To confirm the species of *Anopheles*, molecular identification using PCR was carried out by amplifying the *ITS2* gene for a few randomly selected samples of each species. However, for *Anopheles* mosquitoes from the Leucosphyrus group, all the species were molecularly confirmed. Genomic DNA was extracted from the legs of the mosquitoes using the DNeasy tissue kit (Qiagen, Germany) according to the manufacturer’s protocol. The *ITS2* gene was amplified using primers ITS2A and ITS2B [30]. Each reaction mixture contains 1 × Green GoTaq reaction buffer (Promega), 3.0 mM MgCl_2_ (Promega), 0.2 μM of dNTPs mixture (Promega), 0.5 μM forward and reverse primers, 1 U of GoTaq DNA polymerase (Promega), 5.0 μl of DNA template and sterile dH_2_O up to 25 μl final volume. Cycling parameters consisted of initial denaturation at 95 °C for 2 min, followed by 35 cycles of 95 °C for 30 s, 51 °C for 30 s, 72 °C for 1 min and a final extension at 72 °C for 10 min. The amplified products were excised from the gel and sent for sequencing to First BASE Laboratories Sdn. Bhd. Malaysia.

### *Plasmodium* detection

All *Anopheles* mosquitoes caught were dissected to screen for malaria parasites. The mosquitoes were examined for the presence of sporozoites in the salivary glands and for oocysts in the midgut. The positive samples were preserved in 95% ethanol in centrifuge tubes for genomic DNA extraction. DNA was extracted from the parasite-positive guts and glands using the DNeasy tissue kit (Qiagen, Germany) according to the manufacturer’s protocol. Nested PCR assay was performed targeting the *Plasmodium* small subunit ribosomal RNA (*18S rRNA*) gene to identify human malaria parasites (*Plasmodium falciparum*, *P. malariae*, *P. ovale* and *P. vivax*) and simian *Plasmodium* (*P. coatneyi*, *P. cynomolgi*, *P. fieldi*, *P. inui* and *P. knowlesi*) using genus-specific primers for the nest 1 amplification [31], followed by species-specific primers in the nest 2 amplification [32–34].

PCR amplification reaction for nest 1 assay was performed in a final volume of 50 μl containing 5 μl of DNA template, 1 × Green GoTaq reaction buffer (Promega), 3.0 mM MgCl_2_ (Promega), 0.2 μM of dNTPs mixture (Promega), 0.25 μM of each forward (rPLU1) and reverse (rPLU5) primer and 1.25 U of GoTaq DNA polymerase (Promega). The cycling parameter for nest 1 consisted of initial denaturation at 94 °C for 4 min, followed by 35 cycles of 94 °C for 30 s, 55 °C for 1 min, 72 °C for 1 min and a final extension at 72 °C for 10 min. For each 20 μl of nest 2 PCR amplification, 3 μl of nest 1 PCR amplification product was used as DNA template. The concentrations of reagents used in the nest 2 amplifications were identical to those used in the nest 1 reactions except the final concentration of the GoTaq DNA polymerase (Promega), which was 1.0 U. The PCR condition is also identical to that of the nest 1 amplification except for the annealing temperatures (*P. knowlesi* and *P. inui*: 58 °C; *P. coatneyi* and *P. cynomolgi*: 60 °C; *P. fieldi:* 63 °C). Besides, 4 μl of nest 1 PCR product was also used as DNA template to identify human-specific malaria parasites (*P. falciparum*, *P. vivax*, *P. malariae* and *P. ovale*) using the primers and protocol described by Singh et al. [31]. The amplification products were analyzed using 1.5% agarose gel electrophoresis.

### Data analysis

All the data were analyzed using SPSS version 25 statistical software (IBM, Armonk, NY, USA). *Anopheles* species abundance between different collection methods in the Latin square designed experiment was analyzed using the negative binomial generalized linear mixed models (GLMMs) [35]. The methods of trapping mosquitoes were set as fixed effect and the sampling night as a random effect. Tukey contrasts were used to compare differences in species abundance between trapping methods at α = 0.05. The mean nightly catch of each species of *Anopheles* mosquitoes caught was individually compared between Mosquito Magnet and the “gold standard” HLC using a parametric independent t-test, while a non-parametric test (i.e. Mann-Whitney U) was applied for non-normally distributed data. Besides, to evaluate the association between these two trapping methods, Bland-Altman analysis was used [36]. Bland-Altman analysis provides a graphical approach to illustrate the agreement between two quantitative measurements by constructing limits of agreement, which were calculated using the mean and standard deviation (SD) of the differences between two measurements [37]. In addition, a chi-square test of independence was employed to analyze the difference between the proportion of *Anopheles* mosquitoes caught using HLC and Mosquito Magnet for each sampling location. The level of statistical significance was set at *P* < 0.05 for all tests.

## Results

### Composition of mosquito species

A total of 1082 adult mosquitoes were captured at three sampling locations in a 4 × 4 Latin square designed experiment (Table 1). An additional study carried out in Kem Sri Gading to compare between HLC and Mosquito Magnet yielded another 98 mosquitoes (28 from subfamily Anophelinae while 70 from Culicinae). Overall, five *Anopheles* species were identified in this study (Table 2). The dominant *Anopheles* species varied between sampling areas (Additional file 1: Table S1). In general, the species diversity was very low across all four different sampling locations. Hence, the diversity indices were not calculated. Among all the *Anopheles* mosquitoes, *An. maculatus* was the predominant species, which were mostly collected from Serendah (*n* = 197). All *An. sinensis* (*n* = 183) were collected from Kota Damansara forest reserve while *An. cracens* (*n* = 22) and *An. barbirostris gp.* (*n* = 3) were from Kem Sri Gading. On the other hand, most of the *An. introlatus* were collected from Bukit Tinggi forest (*n* = 98) followed by Kem Sri Gading (*n* = 3) (Table 2).

**Table 1:** Summary of mosquitoes caught by each trap type in the Latin square designed experiment in three different sampling locations in Peninsular Malaysia

Subfamily	HBT	CDC	HLC	MM	Total
Anophelinae	87	13	321	58	479
Culicinae	72	75	354	102	603
Total	159	88	675	160	1082

*HBT* human baited trap, *CDC* CDC light trap, *HLC* human landing catch, *MM* Mosquito Magnet

**Table 2:** Overall number of mosquito species collected from four different sampling locations in Peninsular Malaysia

	HBT	CDC	HLC	MM	Total
*An. barbirostris gp*	0	0	2	1	3
*An. cracens*	0	0	16	6	22
*An. introlatus*	8	3	56	34	101
*An. maculatus*	47	3	142	6	198
*An. sinensis*	32	7	126	18	183
*Aedes*	39	36	209	44	328
*Culex*	28	39	153	71	291
*Armigeres*	2	0	29	19	50
*Mansonia*	3	0	0	1	4
Total	159	88	733	200	1180

*HBT* human baited trap, *CDC* CDC light trap, *HLC* human landing catch, *MM* Mosquito Magnet

### Presence of malaria parasites in *Anopheles* mosquitoes

A total of 8 out of 507 *Anopheles* mosquitoes were positive for malaria parasites, all of which came from the Leucosphyrus group (*An. introlatus* and *An*. *cracens*). All the trapping methods collected *Anopheles* mosquitoes which were positive for simian malaria parasites except the CDC light trap (Table 3). The simian *Plasmodium* detected in the mosquitoes were *P. inui* and *P. fieldi*. None of the mosquitoes were infected with human *Plasmodium* malaria parasites.

**Table 3:** Species of *Plasmodium* identified from the midguts and salivary glands of the *An. introlatus* and *An. cracens* caught using different trapping methods

	Human baited trap (HBT)		Human landing catch (HLC)		Mosquito Magnet (MM)	
	Midgut	Salivary gland	Midgut	Salivary gland	Midgut	Salivary gland
*Plasmodium* species in *An. introlatus*						
* Pin*	0	0	2	1	0	0
* Pfi*	1	0	1	1	0	0
* Pin* + *Pfi*	0	0	1	0	1	0
*Plasmodium* species in *An. cracens*						
Pin	0	0	0	0	0	1
Total	1	0	4	2	1	1

*Pin* *Plasmodium inui*; *Pfi* *Plasmodium fieldi* (1 of the sample was positive for both oocysts and sporozoites)

### *Anopheles* abundance based on trapping methods

**Fig. 3:**
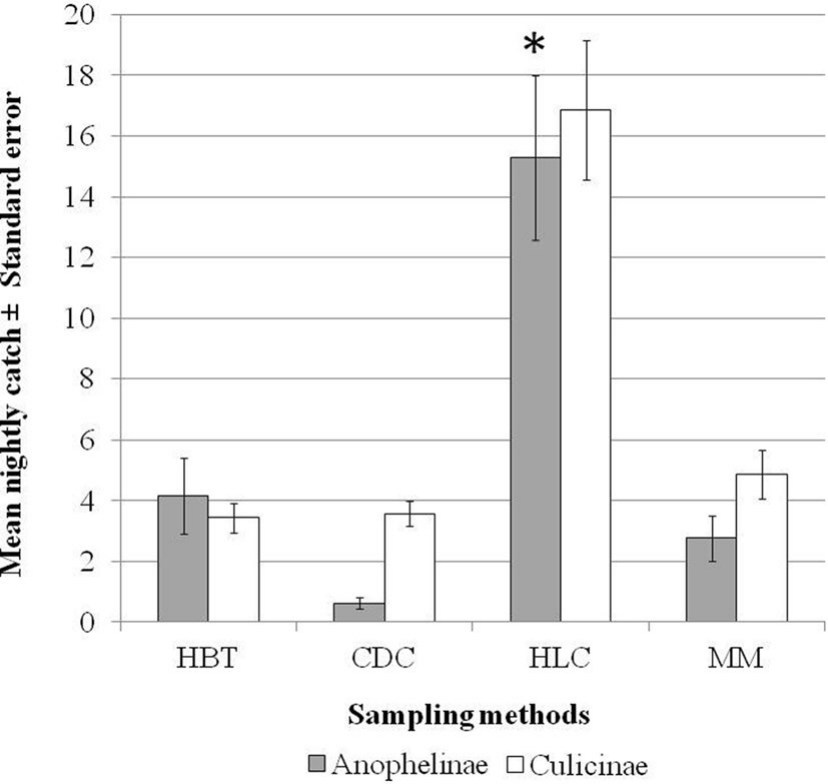
Mean nightly catches (± standard error, SE) of mosquitoes according to subfamily using four different methods in a Latin square designed experiment; *GLMM, *P* < 0.05. HBT: human baited trap; CDC: CDC light trap; HLC: human landing catch; MM: Mosquito Magnet

The Latin square designed experiment which was conducted at the three different locations showed that HLC caught more *Anopheles* mosquitoes (*n* = 321) compared to HBT (*n* = 87), Mosquito Magnet (*n* = 58) and CDC light trap (*n* = 13). The GLMM analysis showed that HLC yielded a significantly higher mean nightly catch of *Anopheles* mosquitoes compared to Mosquito Magnet (*P* = 0.049). However, there was no significant difference in mean nightly catch of the *Anopheles* mosquitoes between Mosquito Magnet and other trapping methods, HBT (*P* = 0.646) and CDC light traps (*P* = 0.197). Similarly, HLC also produced the highest mean nightly catch of mosquitoes from the subfamily Culicinae compared to the other three methods (Fig. 3).

Interestingly, a study at Bukit Tinggi forest in Johor showed that Mosquito Magnet was able to catch a higher percentage of *Anopheles* mosquitoes (34.3%) compared to Kota Damansara (9.8%) and Serendah (3.0%). This finding prompted an additional study at forest site in Kem Sri Gading, Pahang, where a preliminary survey had shown the presence of *An. cracens*.

**Fig. 4:**
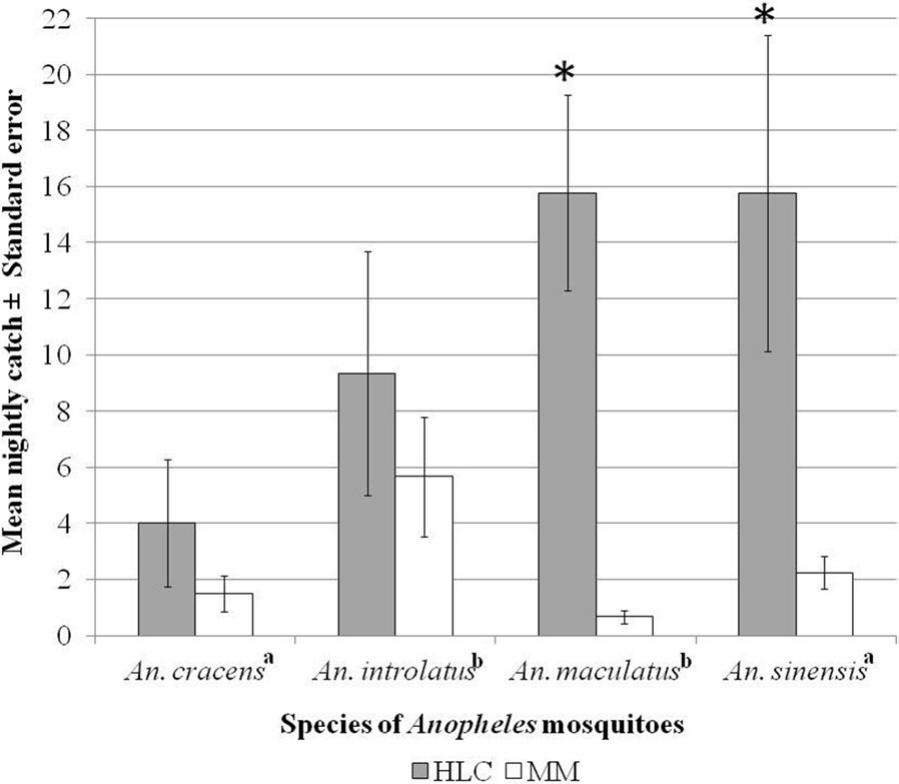
Mean nightly catches (± standard error, SE) of *Anopheles* mosquito species between human landing catch (HLC) and Mosquito Magnet (MM). ^a^Independent t-test and ^b^Mann-Whitney U test, *P* < 0.05 (*)

The data for *An. cracens* and *An. sinensis* were normally distributed. Therefore, an independent t-test was run on the data with a 95% confidence interval (CI) for the mean difference. On the other hand, data for *An. introlatus* and *An. maculatus*, which were not normally distributed, were analyzed using Mann-Whitney U test. Although the mean nightly catch of both *An. introlatus* (9.33 ± 4.341), *U* = 14.0, *Z* = − 0.647, *P* = 0.589, and *An. cracens* (4.00 ± 2.273), *t*_(6)_ = 1.058, *P* = 0.331, caught using HLC was higher than Mosquito Magnet, the difference was not statistically significant (*P* > 0.05). This was in contrast to *An. sinensis* (15.75 ± 5.640), *t*_(7.2)_ = 2.381, *P* = 0.048, and *An. maculatus* (15.78 ± 3.479), *U* = 3.0, *Z* = -3.366, *P* = 0.001, where HLC showed a significantly higher number of caught mosquitoes compared to Mosquito Magnet (*P* < 0.05) (Fig. 4).

### Associations between HLC and Mosquito Magnet for the collection of *Anopheles* from the Leucosphyrus group

**Fig. 5:**
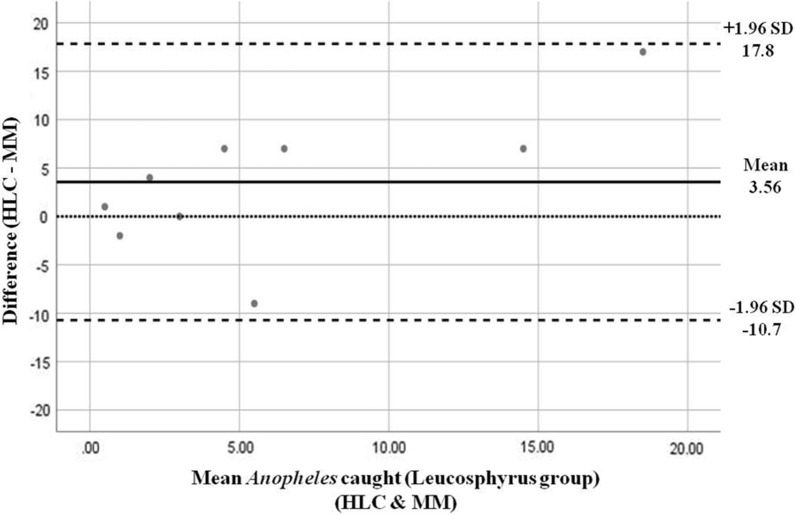
Bland-Altman analysis of *Anopheles* from the Leucosphyrus group caught from human landing catch (HLC) and Mosquito Magnet. The line of equality (dotted line) represents perfect agreement between the two methods. Mean difference (solid line) indicates bias from equality, and limits of agreement are set at ± 1.96 SD of the mean difference (dashed line, s)

Further tests between the HLC and Mosquito Magnet for the *Anopheles* from the Leucosphyrus group using a one-sample t-test showed a non-statistically significant difference between the mean difference of measurement and the test value 0. This shows that there was a certain level of agreement between these two trapping methods. A Bland-Altman plot was constructed to showcase the consistency between the number of *Anopheles* from the Leucosphyrus group caught in HLC and Mosquito Magnet (Fig. 5). The plot shows that all the data fall within the limits of agreement set at ± 1.96 SD of the mean difference (Fig. 5, dashed lines). At lower population density (< 5 mean catches of *Anopheles* per night), the difference between catches using HLC and Mosquito Magnet was smaller, but increased as the density increased. This density-dependent correlation indicates that at a higher mosquito density, HLC was able to capture a greater number of *Anopheles* mosquitoes compared to Mosquito Magnet. The mean difference also indicated that HLC was able to catch three to four *Anopheles* mosquitoes more compared to Mosquito Magnet each night. The line of equality at 0 showed the perfect agreement between the two methods where there was no difference in the catch by both methods. However, since the line of equality 0 falls slightly outside the observed mean, there were differences in the catch between the two methods, where the *Anopheles* catch was slightly biased toward HLC.

### Proportion of *Anopheles* species collected between HLC and Mosquito Magnet

Further statistical analysis was performed using chi-square test of independence to examine the relationship between the trapping methods (HLC and Mosquito Magnet) and the ability to catch *Anopheles* mosquitoes, which were predominant in each sampling location. Interestingly, the proportion of *An. cracens* and *An. introlatus* caught was not significantly affected by the method of catching, i.e. HLC and Mosquito Magnet. There was no significant association between traps used and the proportion of *Anopheles* caught for both *An. introlatus*, χ^2^ = 2.812, *df* = 1, *P* = 0.094, and *An. cracens,* χ^2^ = 2.154, *df* = 1, *P* = 0.142. This indirectly indicates the proportion of *An. cracens* and *An. introlatus* caught by both HLC and Mosquito Magnet is relatively comparable. However, this was different for both *An. maculatus* (χ^2^ = 16.102, *df* = 1, *P* < 0.001) and *An. sinensis* (χ^2^ = 5.102, *df* = 1, *P* = 0.024), where there was a statistically significant association between the proportions of *Anopheles* caught and the methods used. In other words, HLC caught a significantly higher proportion of *Anopheles* mosquitoes compared to Mosquito Magnet for both *An. maculatus* and *An. sinensis* (Table 4).

**Table 4:** Proportion test for the predominant *Anopheles* mosquitoes caught using HLC and Mosquito Magnet according to the study areas

Sampling locations	Date of collection	Predominant *Anopheles* species	HLC	MM	Proportion test (*P* value)
Community forest reserve in Kota Damansara, Selangor	December 2019	*An. sinensis*	126	18	0.024
		Total mosquitoes collected	290	64	
		Proportion	**0.43**	**0.28**	
A small forest patch in Serendah, Selangor	November 2019–December 2019	*An. maculatus*	141	6	< 0.001
		Total mosquitoes collected	320	46	
		Proportion	**0.44**	**0.13**	
Bukit Tinggi forest, Johor	July 2020	*An. introlatus*	53	34	0.094
		Total mosquitoes collected	65	50	
		Proportion	**0.82**	**0.68**	
Forest reserve in Kem Sri Gading, Pahang	Aug 2020 and Oct 2020	*An. cracens*	16	6	0.142
		Total mosquitoes collected	58	40	
		Proportion	**0.28**	**0.15**	

## Discussion

Few studies had previously evaluated different trapping methods for monitoring malaria vectors. These include the conventional trapping methods such as CDC light traps [24], human-baited double net (HDN) traps [25], BG-malaria traps [38, 39] and monkey-baited traps [40] as well as some of the latest methods, including M-Tego [41], Suna traps [42] and electric nets [43]. All these studies agree that different sampling techniques influence the quantity and diversity of the mosquitoes collected. Thus, with the emergence of knowlesi malaria in many countries in Southeast Asia, there is a need for more robust and effective trapping methods which are able to capture the local *Anopheles* mosquitoes particularly from the Leucosphyrus group, which are the vectors for knowlesi malaria [16] and other zoonotic simian *Plasmodium*.

This study to our knowledge represents the first description and comparison of Mosquito Magnet to some of the commonly used mosquito trapping methods to sample *Anopheles* mosquito for malaria studies in Malaysia. Generally, HLC performed best at catching mosquitoes from both the Anophelinae and Culicinae subfamilies. However, HLC has its limitations. Some of the limitations of using HLC include probable variation in the attractiveness of human hosts to mosquitoes due to different body odor [15] and ethical considerations regarding accidental infection with malaria. Detection of simian *Plasmodium* in the salivary glands of some mosquitoes collected in this study highlights the inherent risk of exposure to infectious bites during the collection period using HLC. In addition, there is the probability of interpersonal variation of skills among the collectors, which can indirectly lead to biasness in the collection. On the other hand, Mosquito Magnet, which does not use humans as the natural bait, can be an alternative strategy that allows standardized sampling conditions for the surveillance of exophagic simian malaria vectors.

Contrarily, CO_2_ baited CDC light traps performed very poorly compared to the other three methods. In this study, the CDC light trap caught the fewest of all three *Anopheles* species (*An. sinensis*, *An. introlatus* and *An. maculatus*) obtained from the study sites. A similar study conducted many years ago in a malarious area with high numbers of *An. maculatus* also failed to collect *Anopheles* spp. using CDC light traps [44]. Our findings were also in agreement with the previous study by Rohani et al. (2016) where HLC had a better ability to catch these three species of *Anopheles* mosquitoes compared to CO_2_ baited CDC light traps [24]. However, in their study, *An. cracens* was reported to be more attracted to CDC light traps. Unfortunately, we did not test the CDC light trap in Kem Sri Gading where *An. cracens* were obtained in our study. Both studies had utilized dry ice as the source of CO_2_. Indeed, dry ice has been proven to be more effective in releasing CO_2_, and the efficacy in attracting mosquitoes was significantly higher compared to CO_2_ generated by yeast [45]. However, this is a problem when vector surveillance is conducted in remote areas with no access to dry ice. Nevertheless, this problem can be overcome by the combustion of propane to produce CO_2_ in the Mosquito Magnet. The Mosquito Magnet converts the propane from the gas tank to warm carbon dioxide and moisture through a patented catalytic converter process for the endless release of CO_2_ [22, 26, 27]. The CO_2_ and heat released mimic a human or animal host, which acts as an attractant.

Another alternative method commonly used is the HBT. Previous study in Lao PDR showed that HBT had a similar ability to catch *Anopheles* mosquitoes as HLC but was significantly more efficient than CDC light traps [25]. This was expected since both HLC and HBT use humans as the bait. However, in our studies, HLC caught significantly more *Anopheles* mosquitoes than both the CDC light trap and HBT. These variations are probably due to the differences in the species of *Anopheles* mosquitoes caught in the two studies. Although HBT is an ethically acceptable alternative to HLC as it prevents the collectors from being exposed to mosquitoes and other hematophagous insect bites, it still has an underlying danger of exposing the collectors to dangerous wild animals, a similar risk as with the HLC method. This occurs especially when mosquito collections are carried out in forested areas for long periods of time particularly targeting the vectors of simian malaria. There is also concern that HBT might underestimate the true mosquito abundance as mosquitoes would escape through the gap of the outer net when they cannot feed [46]. However, in our study this probability was reduced by collecting the mosquitoes hourly. Another challenge faced with HBT is the difficulty in finding a suitable place to set up the traps in hilly terrains and deep jungles where there is limited flat ground or cleared space. Areas with dense shrubs and bushes can obstruct the mosquitoes from entering the net through the limited gap in HBTs, which is only few centimeters from the ground. The need for a large clear space to set up the HBT posed another challenge to using this method in forested areas. On the other hand, the Mosquito Magnet requires a very small space to set up, and the usage of portable batteries makes it very easy to station the Mosquito Magnet in any area inside the forest and carry out collections after a certain amount of time.

In this study, the Mosquito Magnet was evaluated to investigate its effectiveness as a trapping method to sample *Anopheles* mosquitoes that can be used in endemic areas of knowlesi malaria. Focusing on the vectors of knowlesi and simian malaria, a further study was carried out to compare the efficiency of Mosquito Magnet in catching *Anopheles* from the Leucosphyrus group of mosquitoes against the “gold standard” HLC. Both the CDC light trap and human baited trap were not further evaluated in Kem Sri Gading because the Latin square designed experiments showed no significant difference in the mean nightly catch of *Anopheles* mosquitoes between the Mosquito Magnet and the other two trapping methods. Of the five *Anopheles* species collected in this study, *An. cracens* [40] and *An. introlatus* [47] are known vectors of knowlesi malaria while *An. maculatus* [28] and *An. sinensis* [48] are known vectors of human malaria. However, to date, *An. sinensis* has not been incriminated as a vector for human malaria in Malaysia. Although Mosquito Magnet did not catch more mosquitoes compared to HLC, the differences were not statistically significant especially for *An. introlatus* and *An. cracens*. Thus, this showed the Mosquito Magnet has a promising ability to catch *An. introlatus* and *An. cracens* compared to *An. sinensis* and *An. maculatus*. On the other hand, no statistical analysis was employed for *An. barbirostris* gp since the number was very low. Statistically, there was a certain level of agreement between HLC and Mosquito Magnet from this study. Thus, Mosquito Magnet has the potential to be used as an alternative tool for vector surveillance for outdoor host-seeking malaria vectors. This is especially for vectors of knowlesi malaria, which were predominantly exophagous and found in relatively high biting rates in farm edges bordering forests and forested areas [49, 50].

Due to its robustness, Mosquito Magnet can be used in outdoor settings. The effectiveness of the Mosquito Magnet also depends on the type of attractant used. In this study, Lurex^3^ attractant was utilized. Other available attractants are Octenol and CO_2_ sachet [51]. A study comparing these different types of attractant revealed that Octenol was more effective in catching mosquitoes compared to the other two attractants [19]. However, that study was conducted in north-central Florida where different species of *Anopheles* were captured: *An. crucians* and *An. quadrimaculatus*. In a separate laboratory experimental study, Lurex^3^ was found to be effective in attracting *An. gambiae* (*s.s.*) [21]. Indeed, different types of attractants or body odor play important roles in catching the different species of host-seeking mosquitoes [17]. This was also shown in our study where the Lurex^3^ attractant used in the Mosquito Magnet might have been a better attractant for *An. cracens* and *An. introlatus* compared to *An. maculatus* and *An. sinensis*. This highlights the importance of testing the Mosquito Magnet with different attractants on the local *Anopheles* vectors before deploying them. Perhaps, it would be worthwhile to try the Octenol attractant in the future to compare the results with Lurex^3^ for vectors of knowlesi malaria in the Southeast Asia setting.

In this study, *P. inui* and *P. fieldi* detected in the *An. introlatus* and *An. cracens*. *Anopheles* from the Leucosphyrus group are known vectors for most simian *Plasmodium* [11]. Unfortunately, no *P. knowlesi* was detected in any of the mosquitoes collected from the four study locations. This might be due to the study locations situated outside the hotspot spatial clusters of knowlesi malaria in Peninsular Malaysia [5]. Besides, both Kem Microwave Bukit Tinggi and Kem Sri Gading Jengka where positive mosquitoes were obtained had only reported sporadic knowlesi malaria cases from 2011 until 2019. The exact locations where the infections might have occurred were uncertain. From the history taking, most likely the infection had occurred while the person traveled deep into the jungle, which was not an ideal location to conduct the Latin square designed experiment because of the risk involved, logistic demands and also the need for clear flat ground to erect the HBT. The high number of *P. inui* detected in the mosquitoes from this study agrees with many other studies conducted in Southeast Asia where *P. inui* and *P. cynomolgi* were more prevalent compared to *P. knowlesi* in both macaques and mosquitoes [52].

There are also some limitations to using Mosquito Magnet for vector surveillance. In this study, the Mosquito Magnet was operated continuously for 6 h. Thus, we were unable to study the biting time of the mosquitoes unlike HLC. However, this limitation can be easily overcome when the net is replaced hourly, depending on the objective of the study. Indeed, it will provide valuable data such as peak biting time that would be comparable to HLC. In addition, Mosquito Magnet might have a limitation in measuring the host preferences of the vectors. The ammonia and lactic acid in the Lurex^3^ attractant used in the Mosquito Magnet act as kairomones to attract anthropophilic *Anopheles* mosquitoes [53, 54]. Thus, it might be a limitation for studies on the vectors which are more attracted to other hosts such as macaques. However, interestingly, the Mosquito Magnet was able to collect more vectors of simian malaria compared to vectors of human malaria. Since macaques are the natural host for *P. knowlesi*, few studies evaluated the efficiency of monkey-baited traps to catch *Anopheles* mosquitoes, especially the vectors of simian malaria. However, those studies showed monkey-baited traps [40] and macaque odor-baited electrocuting nets [43] performed less effectively compared to HLC in trapping the mosquitoes. Perhaps with human populations increasingly encroaching on the macaque habitat, there might be selective pressure for these simian malaria vectors to change their blood meal preference. The usage of macaques for catching mosquitoes for entomological surveillance also poses other challenges such as ethical issues and risk of transmission of *Herpesvirus simiae* and other types of pathogens which are usually present in non-human primates [55]. Besides, usage of monkey-baited traps requires trained personnel to handle the macaques. However, with some minor improvement, a host preference study can be carried out using the Mosquito Magnet. The attractant used in the Mosquito Magnet can be replaced with a small piece of cloth which has been rubbed on the macaques or any other host odor [56]. This will enable host preference studies to be carried out. In addition, caged animals which are potential hosts can also be used together with the Mosquito Magnet in an improvised host decoy trap (HDT) to study host preferences [57].

## Conclusions

This study reveals that HLC remains the best trapping method for catching mosquitoes for vector surveillance. However, the comparable ability of Mosquito Magnet to catch some of the *Anopheles* species makes it an ethical and safer alternative. Mosquito Magnet, which is less labor intensive, can be effectively used to study the vectors of zoonotic simian malaria especially *An. cracens* and *An. introlatus*, which are the vectors of knowlesi malaria in Peninsular Malaysia. Further studies are nevertheless needed to confirm the catching efficiency of Mosquito Magnet on other *Anopheles* species from the Leucosphyrus group. Ideally, the choice of alternate method for the capture of the zoonotic simian malaria vectors must consider the host preference of the vectors and the ability of the method to represent the results of human attraction shown through HLC. However, the cost of the Mosquito Magnet could be a prohibiting factor, but studies should be conducted to show that regular use of Mosquito Magnet can help to reduce vector density. If this can be proven at least there is a tool available for the surveillance and control of *P. knowlesi* vectors.

## Supplementary Information

**Additional file 1: Table S1.** The overall number of mosquito species collected using different trapping methods in four different locations in Peninsular Malaysia.

## Data Availability

The datasets supporting the conclusion of this article are included within the article.

## Abbreviations

CDC: Centers for Disease Control
GLMM: Generalized linear mixed model
HBT: Human baited trap
HLC: Human landing catch
MM: Mosquito Magnet
PCR: Polymerase chain reaction
