# Supplementary material for: Evaluation of Mosquito Magnet and other collection tools for Anopheles mosquito vectors of simian malaria
Source: Parasit Vectors. 2021 Apr 1;14:184. doi: 10.1186/s13071-021-04689-3 (PMC8015311; doi:10.1186/s13071-021-04689-3)
Supplement: Supplementary file 1 — Additional file 1: Table S1. The overall number of mosquito species collected using different trapping methods in four different locations in Peninsular Malaysia. [file 13071_2021_4689_MOESM1_ESM.docx]

**Additional file 1: Table S1.** The overall number of mosquito species collected using different trapping methods in four different locations in Peninsular Malaysia.

|  | Community forest reserve in Kota Damansara, Selangor | | | | A small forest patch in Serendah, Selangor | | | | Bukit Tinggi forest, Johor | | | | Forest reserve in Kem Sri Gading, Pahang | | Total |
| --- | --- | --- | --- | --- | --- | --- | --- | --- | --- | --- | --- | --- | --- | --- | --- |
| Mosquito species | CDC | HBT | HLC | MM | CDC | HBT | HLC | MM | CDC | HBT | HLC | MM | HLC | MM |  |
| *An. barbirostris gp* | 0 | 0 | 0 | 0 | 0 | 0 | 0 | 0 | 0 | 0 | 0 | 0 | 2 | 1 | 3 |
| *An. cracens* | 0 | 0 | 0 | 0 | 0 | 0 | 0 | 0 | 0 | 0 | 0 | 0 | 16 | 6 | 22 |
| *An. introlatus* | 0 | 0 | 0 | 0 | 0 | 0 | 0 | 0 | 3 | 8 | 53 | 34 | 3 | 0 | 101 |
| *An. maculatus* | 0 | 0 | 0 | 0 | 3 | 47 | 141 | 6 | 0 | 0 | 1 | 0 | 0 | 0 | 198 |
| *An. sinensis* | 7 | 32 | 126 | 18 | 0 | 0 | 0 | 0 | 0 | 0 | 0 | 0 | 0 | 0 | 183 |
| *Aedes* | 12 | 8 | 72 | 4 | 19 | 24 | 123 | 23 | 5 | 7 | 6 | 12 | 8 | 5 | 328 |
| *Culex* | 22 | 19 | 87 | 39 | 15 | 7 | 55 | 16 | 2 | 2 | 2 | 4 | 9 | 12 | 291 |
| *Armigeres* | 0 | 0 | 5 | 2 | 0 | 0 | 1 | 1 | 0 | 2 | 3 | 0 | 20 | 16 | 50 |
| *Mansonia* | 0 | 0 | 0 | 1 | 0 | 3 | 0 | 0 | 0 | 0 | 0 | 0 | 0 | 0 | 4 |
| Total | 41 | 59 | 290 | 64 | 37 | 81 | 320 | 46 | 10 | 19 | 65 | 50 | 58 | 40 | 1180 |

Abbreviations: *HBT*, human baited trap; *CDC*, CDC light trap; *HLC*, human landing catch; *MM*, Mosquito Magnet
